# Correlation Between Child's Dental Fear and Anxiety Symptoms and Evaluating Factors Associated With Dental Fear

**DOI:** 10.1002/hsr2.70879

**Published:** 2025-05-26

**Authors:** Pegah Farzanegan, Maryam Ghasempour, Armon Massoodi, Hoda Shirafkan

**Affiliations:** ^1^ Student Research Committee Babol University of Medical Sciences Babol Iran; ^2^ School of Dentistry Babol University of Medical Sciences Babol Iran; ^3^ Oral Health Research Center, Institute of Health Babol University of Medical Sciences Babol I. R. Iran; ^4^ Social Determinants of Health Research Center, Health Research Institute Babol University of Medical Sciences Babol Iran

**Keywords:** child, dental anxiety, fear, pediatric dentistry

## Abstract

**Background and Aims:**

This study aimed to assess the correlation between dental fear and anxiety problems and evaluate factors associated with dental fear.

**Methods:**

In this study, we utilized the standard Persian version of the Children's Fear Survey Schedule‐Dental Subscale (CFSS‐DS) questionnaire and the Persian version of Spence Children's Anxiety Scale (SCAS) questionnaire to explore children's dental fear and anxiety problems, respectively. Questionnaires were completed by 290 children aged 8–12 years who visited Pediatrics Department of Babol Dental Clinic and private pediatric offices. Demographic characteristics were also recorded. The data were analyzed by the one‐way ANOVA, independent *t*‐test (*α* = 0.05), Pearson correlation, and logistic regression test using SPSS version 26.

**Results:**

In this study, 151 boys and 139 girls were included. Based on the CFSS‐DS questionnaire, 8.6% of children had dental fear (Scores > 38). Dental fear correlated with all aspects of anxiety symptoms (*p* < 0.05). There were significant correlations between children's age (*p* = 0.019) and location of dental treatment (*p* = 0.002) with their dental fear scores. Having a housewife mother is a protective factor in child's dental fear (OR = 0.380, CI = 0.989–0.047, and *p* = 0.047).

**Conclusion:**

The findings of the present study suggest that dental fear and anxiety problems correlate with each other, and factors such as child's age, the location of dental treatment, and having a housewife mother serve as prominent factors contributing to dental fear of children.

## 1 Introduction

Dental fear (DF) is considered a behavioral, emotional, and physiological reaction to threatening stimuli and is known as one of the main obstacles to providing dental services [1, 2]. Dental fear is one of the main causes of dental avoidance and may lead to dental avoidance as well as poor oral hygiene in children [3]. Numerous interacting factors, including parental, personal, and environmental factors may contribute to dental fear and anxiety of children in a dental care setting [4]. Dental fear and anxiety are often considered the same terms and both are related to the sense of fear and anxiety during dental procedures. Studies have shown the prevalence of dental fear varies between populations and is considered to be 5.7% to 20.2% in children and adolescents [5, 6, 7, 8].

Many factors can influence dental fear among children and adolescents. Various studies have been conducted to assess relevant factors contributing to dental fear [9, 10, 11]. Child's age is a contributing factor to child's dental fear [11]. Most literature agrees that younger children are more likely to have more dental fear compared to older children [9, 12, 13]. On the contrary, other studies showed no difference in the severity of dental fear among different age categories [14, 15, 16]. Research regarding the correlation between gender and DF, showed contradictory results. Some studies believe that girls have higher level of DF than boys [9, 12, 13]. while other studies indicate more DF in boys than in girls [17]. On the contrary, some studies reported no difference in dental fear in both genders [12, 16, 18, 19]. The socioeconomic background of parents is one of the factors that can affect childhood dental fear. Children of parents with low income, unemployed parents, or parents with low education levels tend to have a higher dental fear [13, 20]. On the other hand, in some reports, children from parents of high education level have severe dental fear. This can be explained by their higher access to information on dental procedures [21]. Dental fear is related to an increased number of siblings [20, 22] Conversely, Perez did not report a significant correlation between dental fear and demographic factors like socioeconomic background, parental age, education, and number of children in the family [23]. It is worth mentioning that other children's fears and anxieties positively correlate with dental fear [24].

Anxiety is defined as a bad feeling which has physical and psychological symptoms and may disrupt a person's normal life. It ranges from a mild unpleasant state to a severe fear [25]. Although anxiety‐related disorders are considered one of the most common problems in children and adolescents, they may be underestimated due to various factors. Children, especially those under the age of seven, are unable to express their feelings appropriately, and their parents may not detect their children's feelings properly [26]. The prevalence of anxiety‐related disorders is between 17% and 21%, with approximately 8% of individuals requiring treatment. The anxiety symptoms may vary from mild to severe. The pattern of anxiety symptoms in preschool children may be the same as symptoms in older children; therefore, mild anxiety disorders are more common in 70% of the general pediatric population [27, 28].

According to the fifth edition of the Diagnostic and Statistical Manual of Mental Disorders (DSM‐5), anxiety symptoms have numerous subtypes, including social anxiety disorder (Social phobia), agoraphobia, generalized anxiety disorder, panic disorder, and separation anxiety disorder [25]. Children with dental fears tend to avoid dental care, so they put their oral health at risk. The prevalence of tooth problems like decayed and extracted teeth, and episodes of toothache is higher than in children without dental fear. Dental fear during childhood and adolescence can extend to adulthood and bring negative implications for their oral health [5, 29]. Raadal et al. reported that over two‐thirds of the children with high dental fear had five or more carious lesions [30].

Due to the controversial results of previous studies assessing the risk factors of dental fear and the lack of studies considering the correlation between dental fear and other anxiety symptoms in children, we aimed to investigate the correlation between dental fear and anxiety symptoms in children aged 8 to 12 years referred to pediatric dental centers.

## 2 Materials and Methods

This cross‐sectional study was done in Babol City (North of Iran) and the study was approved by the Ethics Committee of Babol University of Medical Sciences (Approval no: IR.MUBABOL.HRI.REC.1400.082). A number of 290 children aged 8 to 12 years were enrolled in the present study. Our sampling method was convenience sampling and was conducted over 7 months (October 23, 2021 to May 20, 2022). In the following, 145 participants were selected from patients who came for a routine dental visit at the clinic of the dental school in Babol University of Medical Sciences and 145 other participants were selected from six private pediatric dental offices. The inclusion criteria were 8 to 12 year‐old‐children, along with children whose parents approved their participation.

Children with visual and hearing disorders, impossibility of understanding the interview questions, suffering from interfering physical and mental disorders (including neurological disorders such as metabolic disorders, and cancer), and children with autism spectrum disorder based on previous history were excluded from the study. Therefore, we selected our participants based on their availability in the place, their interest in contributing to our project as well as considering the inclusion and exclusion factors. Demographic factors including age, gender, parental occupation and education level, child's birth order in a family, and history of visiting a dentist were recorded by a checklist before answering the CFSS‐DS questionnaire. Moreover, to evaluate the correlation between dental fear and anxiety problems by age, children were divided into two age categories, including those aged 10 years and younger, and those older than 10 years.

### 2.1 Questionnaires

In this study, we used the standard Persian version of CFSS‐DS questionnaire to assess children's dental fear [31]. Furthermore, we obtained the standard Persian version of Spence questionnaire to evaluate children's anxiety symptoms [32].

CFSS‐DS is a standard questionnaire that contains 15 questions ranging from score 1 (I am not afraid) to score 5 (I am so afraid) and according to the answers, the overall score of each person varies from 15 to 75, and the score equal to or higher than 38 indicates dental fear in a child [31].

Spence questionnaire was the next questionnaire which is standardized for 6‐12‐years‐children in Iran [33]. It consists of 44 questions in six fields (separation anxiety, social phobia, obsessive‐compulsive disorder, panic agoraphobia, generalized anxiety, and fears of physical injury). Children were instructed to rate on a 4‐point Likert scale including never (0), sometimes (1), often (2) and always (3) based on the frequency of each item they experienced. Scores should be interpreted in comparison to standard norms for different age and gender categories because the mean scores tend to be different between both genders as well as different age groups. Therefore, this standardized score is called a T‐score. A T‐score of less than 60 is interpreted as the normal range of anxiety. A T‐score of 60 or above indicates higher‐than‐normal anxiety levels, which is why the term “elevated anxiety” was employed. Parents were asked not to interfere in the process of answering the questions by their children. If any of the questions were not clear, only the child's question was answered and full explanations were given by the researcher.

### 2.2 Statistical Analysis

The data was analyzed using SPSS software version 26. Descriptive analysis was presented using mean and standard deviation (for quantitative data). Independent sample *t*‐test, ANOVA, and Pearson correlation (*r*) tests were used to check the differences and correlations between variables. The researchers utilized multivariable logistic regression to examine the variables that influence dental fear. To estimate the regression coefficients, we used the maximum likelihood stepwise method. Crude and adjusted odds ratios (OR) and their 95% confidence interval (95% CI) were reported. A *p*‐value of 0.05 was statistically considered significant. In our study, Dental fear was an independent variable and child's age and gender, parental education and occupation, birth order of children, history of visiting a dentist and location of dental treatment were dependent variables.

## 3 Results

A total of 290 children participated in the study, consisting of 151 boys (52.1%) and 139 girls (47.9%), with a mean age of 9.75 ± 1.38 years. In this study, the prevalence of participants experiencing dental fear was evaluated 8.6% (CFSS‐DS > 38) with the lowest score of 15 and the highest score of 60. The results showed 86.6% of children had normal anxiety, while 13.4% had elevated anxiety. The mean of T‐scores was higher in boys than in girls (Boys: 51.52 ± 9.824 vs. girls: 46.21 ± 9.834) and this difference was statistically significant (*p* < 0.05). The results showed a significant moderate correlation between dental fear and anxiety symptoms (*r* = 0.384, *p* < 0.001).

Based on the results presented in Table 1, there were significant correlations between dental fear and child's age (*p* = 0.019) as well as location of the dental treatment (clinic or office) (*p* = 0.002).

**Table 1:** Mean dental fear scores in children in relation to demographic variables.

Dependent variables	Options	mean ± SD dental fear	*p*‐value
Child gender	Girl	24.50 ± 8.747	0.257^a^
	Boy	25.71 ± 9.43	
Child age	Age 8 to 10	25.75 ± 9.72	**0.019** b
	Age more than 10 to 12	23.23 ± 7.41	
Mother's education level	Illiterate	27.00 ± 10.39	0.113^b^
	High school	22.55 ± 8.51	
	Diploma	25.21 ± 8.80	
	Academic	26.25 ± 9.608	
father's education level	Illiterate	18.80 ± 7.43	0.536^b^
	High school	25.25 ± 9.03	
	Diploma	24.87 ± 9.43	
	Academic	25.43 ± 9.070	
Mother's occupation	Housewife	24.60 ± 8.81	0.099^a^
	Employed	26.62 ± 9.83	
Father's occupation	Self‐employed	25.08 ± 9.17	0.858^a^
	civil servant	25.30 ± 9.01	
History of visiting a dentist	Yes	24.92 ± 8.69	0.405^a^
	no	26.90 ± 12.28	
Birth order	First	25.96 ± 9.28	0.133^b^
	Second	24.23 ± 8.91	
	Third	22.00 ± 8.31	
Location	Office	23.43 ± 8.96	**0.002** a
	Clinic	26.73 ± 8.99	

a Independent sample *t*‐test.

b ANOVA, Bold = *p* < 0.05.

According to Table 1, dental fear decreases with each subsequent birth order in the family; however, this trend was not statistically significant (*p* > 0.05). No significant correlation was found between dental fear and other parameters, such as child gender, parental education and occupation, and the history of visiting a dentist (*p* > 0.05).

We entered all demographic and independent variables in multivariable logistic regression. Eventually, in step 15, three variables remained, including mother's occupation, generalized anxiety disorder (GAD) and fear of physical injury. As shown in Table 2, the children whose mothers were housewives, had less dental fear in comparison with children whose mothers were employed (OR = 0.380, CI = 0.989–0.047, and *p* = 0.047). In children with GAD, a one‐point increase in GAD's score is associated with a 25% increase in the likelihood of experiencing dental fear. (OR = 1.250, CI = 1.100–1.421, and *p* = 0.006). In addition, among children with a fear of physical injury, a one‐unit increase in the fear of injury score is associated with a 26% increase in the likelihood of experiencing dental fear (OR = 1.266, CI = 1.092–1.421, and *p* = 0.002).

**Table 2:** Investigating effect of demographic factors and anxiety symptoms on dental fear.

Variables		Crude OR	CI		*p*‐value	Adjusted OR	CI		*p*‐value
			Lower	Upper			Lower	Upper	
Gender (male)		1.329	0.484	3.651	0.581	—	—	—	—
Age		0.990	0.324	3.024	0.987	—	—	—	—
Mother's level of education^a^	Uneducated	0.000	0.000	—	0.999	—	—	—	—
	High school	0.512	0.052	5.010	0.565	—	—	—	—
	Diploma	1.567	0.404	6.078	0.516	—	—	—	—
Father's level of education^b^	Uneducated	0.000	0.000	—	0.999	—	—	—	—
	High school	0.529	0.095	2.940	0.467	—	—	—	—
	Diploma	0.529	0.084	1.979	0.265	—	—	—	—
Father's occupation^c^:	Self‐employment	1.520	0.420	5.500	0.523	—	—	—	—
Mother's occupation^d^:	Housewife	0.515	0.139	1.914	0.322	0.380	0.146	0.989	0.047
History of visiting a dentist		0.380	0.085	1.701	0.206	—	—	—	—
Reason for visiting a Dentist		0.584	0.170	2.014	0.395	—	—	—	—
Separation anxiety disorder		1.054	0.890	1.248	0.543	—	—	—	—
Social anxiety disorder		0.934	0.786	1.110	0.440	—	—	—	—
Obsessive‐compulsive disorder		0.898	0.740	1.090	0.277	—	—	—	—
General anxiety disorder		1.402	1.102	1.784	0.006	1.250	1.100	1.421	0.001
Agoraphobia		0.958	0.803	1.144	0.637	—	—	—	—
Fear of physical injury		1.289	1.069	1.555	0.008	1.266	1.092	1.421	0.002

^a^ Bachelor's and higher considered as reference category.

^b^ Bachelor's and higher considered as reference category.

^c^ Employee considered as reference category.

^d^ Employed considered as reference category.

## 4 Discussion

Despite the implementation of pain control techniques in dental offices and training for dentists on building trust with patients, dental fear continues to be a substantial issue in the treatment of children and teenagers [25]. Children's fear of dental procedures can lead to long‐term consequences [34]. Dental fear can be costly for families and society and even endanger the physical and mental health of patients, especially children [35]. Childhood anxiety can act as one of the effective factors in the occurrence of defensive fear reactions in the dental environment. A child's anxiety can originate from the surrounding environment, such as family factors, friends, society and previous stimuli [5]. Therefore, the aim of this study was to determine the role of childhood anxiety and demographic characteristics in the occurrence of dental fear.

In this study, the mean score of CFSS‐DS questionnaire was higher than in previous studies [5, 36, 37]. However, the mean score of dental fear in this study was lower than similar studies which include the study by Varmazyar et al. done in a number of 185 children between the ages of 6 to 12 years [38] as well as the study done by Andrade et al. among Brazilian children aged 8 to 10 years [39]. Differences between the results of similar studies may be due to cultural background of the participants or the less use of oral care services in some developing countries.

### 4.1 Correlation Between Dental Fear and Anxiety Symptoms

In the current research, a significant correlation was found between dental fear and anxiety symptoms which is in agreement with the findings of Kronina et al. [17, 24]. Anxiety problems in children may affect children's behavior in dental settings. Therefore, anxiety can be considered a factor contributing to child's dental fear. Furthermore, children with anxiety problems who reveal behavior management problems should be considered patients at risk of developing dental fear, Therefore, familiarizing them with all new treatment tools and procedures is crucial for effectively managing their anxiety symptoms [24, 40].

### 4.2 Correlation Between Age and Dental Fear in Children

There was a significant correlation between children's age and their dental fear in the current research. The data in shows that as children grow older, their average dental fear scores decrease. Other research studies have found similar results [5, 17, 41, 42, 43]. They reported that with decreasing age, the level of dental fear increases significantly, which was consistent with our findings. Contrary to these results, some other studies have found that dental fear tends to rise as individuals get older [10, 44]. Interestingly, some other reports found no significant correlation between dental fear and age of children [14, 45]. In many studies, age is considered one of the main factors affecting child's fear [46, 47]. As children grow older, the connection between fear and age weakens due to the strengthening of the Ego and cognitive abilities, enabling them to better predict, understand, and manage emotions. As children grow older the correlation between fear and children's age weakens due to the strengthening of the Ego and cognitive ability, enabling them to understand, predict better, and manage emotions [5]. This ability makes the child more flexible in dealing with frightening stimuli [9]. Contrary results of different studies can be due to some factors such as previous painful dental treatment [48].

### 4.3 Correlation Between Gender and Dental Fear in Children

This study found no significant correlation between fear scores in children and their gender, which aligns with the results of similar studies [5, 38, 49]. However, contrary to these findings, in other studies, girls had significantly higher dental fear than boys [50, 51, 52] and in a study on the age group of 9 to 11 years, boys were more afraid of dental treatment than girls [53], which is inconsistent with our findings. Differences between the results of similar studies can be due to the cultural background of the participants, biological differences between boys and girls and the scale used for measuring dental fear [5].

### 4.4 Correlation Between the History of Visiting a Dentist and Dental Fear in Children

In our study, the average fear scores of children who had previously visited a dentist were lower than those of children who had not, though this difference was not statistically significant. In a similar study, they reported higher dental fear in children without a history of visiting a dentist which is in agreement with our findings [2]. Children with past dental visits were more cooperative and had lower dental fear compared to children who had not visited a dentist before. Having more dental fear in children without previous dental visits can be due to their incorrect insights about dental procedures before visiting a dentist, so past dental history can be an indicator of dental fear in children [54, 55]. It is worth mentioning that aggressive dental treatments in the first visits may negatively impact children's behavior during subsequent visits so they may not cooperate well with their dentist. Nevertheless, it is recommended to have oral examinations and prophylaxis for the first visits rather than having aggressive treatments like tooth extractions [2].

### 4.5 Correlation Between Birth Order and Dental Fear in Children

In the present study, dental fear decreases by increasing the child's order in the family but this difference is not statistically significant. On the contrary, a study by Aminabadi et al. [56] reported that dental fear in first‐born children is lower than in second‐born children and dental fear increases by increasing the child's order in the family which is inconsistent with our findings. Moreover, Ghaderi et al. [57] reported middle children have the least dental fear compared to other birth orders of children in the family. Another study revealed that there was no correlation between dental fear and the order in which children are born in a family [58]. Having more dental fear in first‐born children compared to other birth orders of children in the family can be due to the fact that first‐born children may receive more attention and care within the family, which may explain this phenomenon. They are usually vulnerable to stressful situations, so they may not be able to cope with their negative feelings in dental settings and they have more dental fear compared to their other siblings. The atmosphere of the family changes with the birth of a new child. As a result, children's personalities tend to develop more positively through interactions with their siblings. First‐born children, having spent more time alone, acquire social skills later than their younger siblings, which may explain why they experience higher levels of dental fear compared to their second and third‐born counterparts. While variations in study designs and methods of data collection may contribute to the conflicting findings, biological factors also play a significant part in shaping children's nature and influencing the outcomes of comparable research studies [57].

### 4.6 Correlation Between Location of Dental Treatment and Dental Fear in Children

The current research found a strong correlation between children's dental fear and the location of dental treatment. Children who were treated at dental pediatric offices had higher dental fear compared to children treated at dental clinics. Treatment costs in pediatric dental offices are usually higher than dental clinics and families with higher socioeconomic status usually prefer to visit dental offices rather than dental clinics. In other words, families of higher social levels are usually more educated and more conservative about issues related to health, so they may unconsciously transfer their emotions and concerns to their children. Therefore, their children may end up having more dental fear compared to children treated at dental clinics who are mostly from families of lower social level and may not have much knowledge and concerns about dental treatments. Our findings was consistent with consistent with Sindhu et al. study; they related lower DF among patients with lower socioeconomic status to the differences in social and cultural factors as well as lower perception and higher patience among deprived patients which leads to lower DF [59]. Conversely, Krishnamurthy et al. found that patients with lower socioeconomic status higher DF [60]. Patients from lower socioeconomic status deal with physical and psychological problems and they have limited access to resources. Therefore, they end up having more DF compared to children from higher socioeconomic background. These controversy results can be due to differences in study population and their cultural and behavioral differences as well as a bad dental experience of children, no matter of their socioeconomic status, can increase their DF.

### 4.7 Investigating the Effect of Demographic Factors and Anxiety Symptoms on Dental Fear

In the current study's regression model, being a housewife mother is a protective factor for dental fear of their children. This can be due to the fact that housewife mothers can allocate more time to their children, so their children can gain profitable skills to deal with stressful situations. Consequently, they have less dental fear compared to children whose mothers are employed and unable to spend proper time with their children.

In the regression model of this study, GAD and fear of physical injury in children are risk factors for dental fear. This can be due to the fact that GAD is characterized by a combination of fears. Any situation perceived as threatening can trigger anxiety intense enough to incapacitate individuals with GAD. Since dental visits often evoke various negative emotions and fears in these patients, they are likely to experience greater dental anxiety compared to those without GAD. Fear of physical injury is defined as a fear of being hurt by different things. For example, when children with a fear of physical injury undergo tooth extraction, they are terrified of losing all their teeth without being replaced by permanent teeth and because dental treatments may involve invasive procedures, children who fear physical injury tend to exhibit dental anxiety, which can lead to a lack of cooperation with their dentist.

## 5 Strength and Limitations

Our study has made a comprehensive conclusion regarding dental fear and anxiety symptoms and the effect of various background variables on DF which is the strength of our study. We used a large sample size in our study to include more children and increasing the power of study while reducing the potential to bias. Our findings rely on a convenience sample, which may not fully represent the broader population. Convincing parents and children to participate in the study was also among the challenges of our study. While our study provides novel insights into correlation between child's dental fear and anxiety symptoms and factors associated with dental fear, these limitations highlight the need for further research with longitudinal designs.

## 6 Conclusion

The present study revealed dental fear score obtained from the CFSS‐DS questionnaire was higher than studies conducted in developing countries like Bosnia and India. Factors such as the age of the child, the child's anxiety problems, the birth order of the child in the family, the location of dental treatment, and having a housewife mother can serve as important factors contributing to child's dental fear. Therefore, increasing our knowledge and information in this field can help us reduce children's dental fear and prevent the negative consequences in the long term.

## Author Contributions

**Pegah Farzanegan:** writing – original draft, writing – review and editing, project administration, resources. **Maryam Ghasempour:** conceptualization, supervision, validation, writing – original draft, writing – review and editing, project administration, resources. **Armon Massoodi:** conceptualization, project administration, supervision. **Hoda Shirafkan:** project administration, data curation, formal analysis, methodology.

## Disclosure

All authors have read and approved the final version of the manuscript. Maryam Ghasempour have full access to all of the data in this study and takes complete responsibility for the integrity of the data and the accuracy of the data analysis.

## Ethics Statement

This study was approved by the ethical committee of Babol University of Medical Sciences (Approval no: IR.MUBABOL.HRI.REC.1400.082).

## Consent

All methods were carried out in accordance with relevant guidelines and regulations. Identified images or other personal or clinical details are not presented in this manuscript.

## Conflicts of Interest

The authors declare no conflicts of interest.

## Transparency Statement

The lead author Maryam Ghasempour affirms that this manuscript is an honest, accurate, and transparent account of the study being reported; that no important aspects of the study have been omitted; and that any discrepancies from the study as planned (and, if relevant, registered) have been explained.

## Data Availability

The data used to support the findings of this study were supplied by corresponding author under license and data will be available on request. Requests for access to these data should be made to corresponding author.

## References

[1] M. J. Y. Yon , K. J. Chen , S. S. Gao , D. Duangthip , E. C. M. Lo , and C. H. Chu , “Dental Fear and Anxiety of Kindergarten Children in Hong Kong: A Cross‐Sectional Study,” International Journal of Environmental Research and Public Health 17, no. 8 (2020): 2827. 32325972 10.3390/ijerph17082827 PMC7215591

[2] M. A. Alshoraim , A. A. El‐Housseiny , N. M. Farsi , O. M. Felemban , N. M. Alamoudi , and A. A. Alandejani , “Effects of Child Characteristics and Dental History on Dental Fear: Cross‐Sectional Study,” BMC Oral Health 18, no. 1 (2018): 33. 29514657 10.1186/s12903-018-0496-4 PMC5842627

[3] A. J. Kvesić , M. Hrelja , Ž. Lovrić , et al., “Possible Risk Factors for Dental Fear and Anxiety in Children Who Suffered Traumatic Dental Injury,” Dentistry Journal 11, no. 8 (2023): 190. 37623286 10.3390/dj11080190 PMC10453853

[4] A. E. Carter , “Pathways of Fear and Anxiety in Dentistry: A Review,” World Journal of Clinical Cases 2, no. 11 (2014): 642. 25405187 10.12998/wjcc.v2.i11.642 PMC4233415

[5] S. Z. Mohebbi , S. Razeghi , M. Gholami , M. J. Kharazifard , and S. Rahimian , “Dental Fear and Its Determinants in 7‐11‐year‐old Children in Tehran, Iran,” European Archives of Paediatric Dentistry 20, no. 5 (2019): 393–401. 30565154 10.1007/s40368-018-0407-z

[6] B. M. Grisolia , A. P. P. Dos Santos , I. M. Dhyppolito , H. Buchanan , K. Hill , and B. H. Oliveira , “Prevalence of Dental Anxiety in Children and Adolescents Globally: A Systematic Review With Meta‐Analyses,” International Journal of Paediatric Dentistry 31, no. 2 (2021): 168–183. 33245591 10.1111/ipd.12712

[7] S. Cianetti , G. Lombardo , E. Lupatelli , et al., “Dental Fear/Anxiety Among Children and Adolescents. A Systematic Review,” European Journal of Paediatric Dentistry 18, no. 2 (2017): 121–130. 28598183 10.23804/ejpd.2017.18.02.07

[8] Y.‐S. Shim , A.‐H. Kim , E.‐Y. Jeon , and S.‐Y. An , “Dental Fear & Anxiety and Dental Pain in Children and Adolescents: A Systemic Review,” Journal of Dental Anesthesia and Pain Medicine 15, no. 2 (2015): 53–61. 28879259 10.17245/jdapm.2015.15.2.53 PMC5564099

[9] Y. S. Shim , A. H. Kim , E. Y. Jeon , and S. Y. An , “Dental Fear & Anxiety and Dental Pain in Children and Adolescents; a Systemic Review,” Journal of Dental Anesthesia and Pain Medicine 15, no. 2 (2015): 53–61. 28879259 10.17245/jdapm.2015.15.2.53 PMC5564099

[10] A. Akbay Oba , Ç. T. Dülgergil , and I. Şaroğlu Sönmez , “Prevalence of Dental Anxiety in 7‐ to 11‐Year‐Old Children and Its Relationship to Dental Caries,” Medical Principles and Practice 18, no. 6 (2009): 453–457. 19797921 10.1159/000235894

[11] P. Busato , R. R. Garbín , C. N. Santos , L. R. Paranhos , and L. Rigo , “Influence of Maternal Anxiety on Child Anxiety During Dental Care: Cross‐Sectional Study,” Sao Paulo Medical Journal 135, no. 2 (2017): 116–122. 28423066 10.1590/1516-3180.2016.027728102016 PMC9977341

[12] M. Kakkar , A. Wahi , R. Thakkar , I. Vohra , and A. K. Shukla , “Prevalence of Dental Anxiety in 10‐14 Years Old Children and Its Implications,” Journal of Dental Anesthesia and Pain Medicine 16, no. 3 (2016): 199–202. 28884153 10.17245/jdapm.2016.16.3.199 PMC5586557

[13] T. Talo Yildirim , S. Dundar , A. Bozoglan , et al., “Is There a Relation Between Dental Anxiety, Fear and General Psychological Status?,” PeerJ 5 (2017): e2978. 28229019 10.7717/peerj.2978 PMC5314953

[14] M. Agarwal and U. Das , “Dental Anxiety Prediction Using Venham Picture Test: A Preliminary Cross‐Sectional Study,” Journal of Indian Society of Pedodontics and Preventive Dentistry 31, no. 1 (2013): 22–24. 23727738 10.4103/0970-4388.112397

[15] K. N. Arapostathis , T. Coolidge , D. Emmanouil , and N. Kotsanos , “Reliability and Validity of the Greek Version of the Children's Fear Survey Schedule‐Dental Subscale,” International Journal of Paediatric Dentistry 18, no. 5 (2008): 374–379. 18284471 10.1111/j.1365-263X.2007.00894.x

[16] A. Rajwar and M. Goswami , “Prevalence of Dental Fear and Its Causes Using Three Measurement Scales Among Children in New Delhi,” Journal of Indian Society of Pedodontics and Preventive Dentistry 35, no. 2 (2017): 128–133. 28492191 10.4103/JISPPD.JISPPD_135_16

[17] G. Klingberg , U. Berggren , S. G. Carlsson , and J. G. Noren , “Child Dental Fear: Cause‐Related Factors and Clinical Effects,” European Journal of Oral Sciences 103, no. 6 (1995): 405–412. 8747678 10.1111/j.1600-0722.1995.tb01865.x

[18] M. Saatchi , M. Abtahi , G. Mohammadi , M. Mirdamadi , and E. S. Binandeh , “The Prevalence of Dental Anxiety and Fear in Patients Referred to Isfahan Dental School, Iran,” Dental Research Journal 12, no. 3 (2015): 248–253. 26005465 PMC4432608

[19] P. Singh , R. Pandey , A. Nagar , and K. Dutt , “Reliability and Factor Analysis of Children's Fear Survey Schedule‐Dental Subscale in Indian Subjects,” Journal of Indian Society of Pedodontics and Preventive Dentistry 28, no. 3 (2010): 151–155. 21157045 10.4103/0970-4388.73788

[20] J. Abanto , E. A. Vidigal , T. S. Carvalho , S. N. C. Sá , and M. Bönecker , “Factors for Determining Dental Anxiety in Preschool Children With Severe Dental Caries,” Brazilian oral research 31 (2017): e13. 28099579 10.1590/1807-3107BOR-2017.vol31.0013

[21] M. L. Goettems , T. M. Ardenghi , A. R. Romano , F. F. Demarco , and D. D. Torriani , “Influence of Maternal Dental Anxiety on the Child's Dental Caries Experience,” Caries Research 46, no. 1 (2012): 3–8. 22156724 10.1159/000334645

[22] L. Merdad and A. A. El‐Housseiny , “Do Children's Previous Dental Experience and Fear Affect Their Perceived Oral Health‐Related Quality of Life (OHRQoL)?,” BMC Oral Health 17, no. 1 (2017): 47. 28093086 10.1186/s12903-017-0338-9 PMC5240375

[23] B. Peretz , Y. Nazarian , and E. Bimstein , “Dental Anxiety in a Students' Paediatric Dental Clinic: Children, Parents and Students,” International Journal of Paediatric Dentistry 14, no. 3 (2004): 192–198. 15139954 10.1111/j.1365-263X.2004.00545.x

[24] L. Kroniņa , M. Rasčevska , and R. Care , “Psychosocial Factors Correlated With Children's Dental Anxiety,” Stomatologija 19, no. 3 (2017): 84–90. 29339671

[25] N. Pop‐Jordanova , “Different Clinical Expression of Anxiety Disorders in Children and Adolescents: Assessment and Treatment,” Prilozi/Makedonska Akademija Na Naukite I Umetnostite, Oddelenie Za Biološki I Medicinski Nauki = Contributions/Macedonian Academy of Sciences and Arts, Section of Biological and Medical Sciences 40, no. 1 (2019): 5–40. 10.2478/prilozi-2019-0001 31152643

[26] B. López‐Pérez and E. L. Wilson , “Parent–Child Discrepancies in the Assessment of Children's and Adolescents' Happiness,” Journal of Experimental Child Psychology 139 (2015): 249–255. 26138699 10.1016/j.jecp.2015.06.006

[27] M. Al‐Biltagi , “Anxiety Disorder in Children: Review,” Journal of Paediatric Care Insight 1, no. 1 (2016): 18–28.

[28] J. S. Comer , K. P. Gallo , P. Korathu‐Larson , D. B. Pincus , and T. A. Brown , “Specifying Child Anxiety Disorders Not Otherwise Specified in the DSM‐IV,” Depression and Anxiety 29, no. 12 (2012): 1004–1013. 22807226 10.1002/da.21981 PMC4258529

[29] F. Anbari , Z. Elmi , F. Anbari , and K. Rezaeifar , “General Anxiety and Dental Fear: Is There a Relationship?,” Journal of Dental Materials & Techniques 8, no. 4 (2019): 190–196.

[30] M. Raadal , G. V. Strand , E. C. Amarante , and G. Kvale , “Relationship Between Caries Prevalence at 5 Years of Age and Dental Anxiety at 10,” European Journal of Paediatric Dentistry 3, no. 1 (2002): 22–26. 12871013

[31] S. Safari , M. Gholami , and S. Razeghi , “Development of a Persian Version of the Children's Fear Survey Schedule‐Dental Subscale (CFSS‐DS) Among 8‐12 Year‐Old Female Students in Tehran,” Journal of Dental Medicine 31, no. 2 (2018): 98–108.

[32] S. H. Spence , “A Measure of Anxiety Symptoms Among Children,” Behaviour Research and Therapy 36, no. 5 (1998): 545–566. 9648330 10.1016/s0005-7967(98)00034-5

[33] R. Mousavi , A.‐R. Moradi , V. Farzad , and S. Mahdavi , “Psychometric Properties of the Spence Children's Anxiety Scale With an Iranian Sample,” International Journal of Psychology 1, no. 1 (2007): 17–26.

[34] S. J. Lindsay , G. Humphris , and G. J. Barnby , “Expectations and Preferences for Routine Dentistry in Anxious Adult Patients,” British Dental Journal 163, no. 4 (1987): 120–124. 3477264 10.1038/sj.bdj.4806218

[35] M. Themessl‐huber , R. Freeman , G. Humphris , S. MacGillivray , and N. Terzi , “Empirical Evidence of the Relationship Between Parental and Child Dental Fear: A Structured Review and Meta‐Analysis,” International Journal of Paediatric Dentistry 20, no. 2 (2010): 83–101. 20384823 10.1111/j.1365-263X.2009.00998.x

[36] J. B. Krikken , A. J. van Wijk , J. M. ten Cate , and J. S. J. Veerkamp , “Measuring Dental Fear Using the CFSS‐DS. Do Children and Parents Agree?,” International Journal of Paediatric Dentistry 23, no. 2 (2013): 94–100. 22339783 10.1111/j.1365-263X.2012.01228.x

[37] P. Wogelius , S. Poulsen , and H. Toft Sørensen , “Prevalence of Dental Anxiety and Behavior Management Problems Among Six to Eight Years Old Danish Children,” Acta Odontologica Scandinavica 61, no. 3 (2003): 178–183. 12868693 10.1080/00016350310003468

[38] A. Varmazyar , S. Taram , and Z. R. Rohani , “Dental Fear of Children and Its Relationship With Caries Experience,” Journal Dental School; Vol 39, no. 1 (2021): 28–32.

[39] N. Andrade , I. Laureano , L. Farias , L. Fernandes , C. Prates , and A. Cavalcanti , “Moderate and High Dental Fear by Sex and Age Using the Children's Fear Survey Schedule‑Dental Subscale,” Revista Portuguesa de Estomatologia, Medicina Dentária e Cirurgia Maxilofacial 62, no. 3 (2021): 157–162.

[40] G. Klingberg , “Dental Fear and Behavior Management Problems in Children. A Study of Measurement, Prevalence, Concomitant Factors, and Clinical Effects,” Swedish Dental Journal Supplement 103 (1995): 1–78. 7740439

[41] M. Paryab and M. Hosseinbor , “Dental Anxiety and Behavioral Problems: A Study of Prevalence and Related Factors Among a Group of Iranian Children Aged 6–12,” Journal of Indian Society of Pedodontics and Preventive Dentistry 31, no. 2 (2013): 82–86. 23886717 10.4103/0970-4388.115699

[42] M. ten Berge , J. S. J. Veerkamp , J. Hoogstraten , and P. J. M. Prins , “Childhood Dental Fear in the Netherlands: Prevalence and Normative Data,” Community Dentistry and Oral Epidemiology 30, no. 2 (2002): 101–107. 12000350 10.1034/j.1600-0528.2002.300203.x

[43] E. Townend , G. Dimigen , and D. Fung , “A Clinical Study of Child Dental Anxiety,” Behaviour Research and Therapy 38, no. 1 (2000): 31–46. 10645022 10.1016/s0005-7967(98)00205-8

[44] K. Rantavuori , M. Tolvanen , H. Hausen , S. Lahti , and L. Seppä , “Factors Associated With Different Measures of Dental Fear Among Children at Different Ages,” Journal of Dentistry for Children (Chicago, Ill) 76, no. 1 (2009): 13–19. 19341574

[45] S. Sathyaprasad , S. Lalugol , and J. George , “Prevalence of Dental Anxiety and Associated Factors Among Indian Children,” Pesquisa Brasileira em Odontopediatria e Clínica Integrada 18, no. 1 (2018): 1–10.

[46] J. Dean and J. Nichol , History 7‐11: Developing Primary Teaching Skills (Routledge, 2002).

[47] K. Sylva , E. Melhuish , P. Sammons , I. Siraj‐Blatchford , and B. Taggart , *Final Report From the Primary Phase: Pre‐School, School and Family Influences on Children's Development During Key Stage*, vol. 2, 7–11 2008.

[48] R. K. Zighair and Z. J. Jafar , “Evaluation of Children's Dental Anxiety in Colorful Versus Conventional Dental Clinic,” International Medical Journal 28, no. 1 (2021): 58–60.

[49] R. Rafatjou , M. Ahmadpanah , B. Ahmadi , and M. Mahmoodi , “Evaluation of Dental Anxiety and the Role of Concomitant Factors in Their Anxiety Level in 9–12 Years Old Children,” Avicenna Journal of Dental Research 11, no. 2 (2019): 53–60.

[50] M. I. Cuthbert and B. G. Melamed , “A Screening Device: Children at Risk for Dental Fears and Management Problems,” ASDC Journal of Dentistry for Children 49, no. 6 (1982): 432–436. 6960031

[51] P. Murray , A. Liddell , and J. Donohue , “A Longitudinal Study of the Contribution of Dental Experience to Dental Anxiety in Children Between 9 and 12 Years of Age,” Journal of Behavioral Medicine 12, no. 3 (1989): 309–320. 2634106 10.1007/BF00844874

[52] J. Olak , M. Saag , S. Honkala , et al., “Children's Dental Fear in Relation to Dental Health and Parental Dental Fear,” Stomatologija 15, no. 1 (2013): 26–31. 23732827

[53] G. Klingberg and A. G. Broberg , “Dental Fear/Anxiety and Dental Behaviour Management Problems in Children and Adolescents: A Review of Prevalence and Concomitant Psychological Factors,” International Journal of Paediatric Dentistry 17, no. 6 (2007): 391–406. 17935593 10.1111/j.1365-263X.2007.00872.x

[54] K. M. Milsom , M. Tickle , G. M. Humphris , and A. S. Blinkhorn , “The Relationship Between Anxiety and Dental Treatment Experience in 5‐year‐old Children,” British Dental Journal 194, no. 9 (2003): 503–506; discussion 495. 12835786 10.1038/sj.bdj.4810070

[55] E. Nicolas , M. Bessadet , V. Collado , P. Carrasco , V. Rogerleroi , and M. Hennequin , “Factors Affecting Dental Fear in French Children Aged 5‐12 Years,” International Journal of Paediatric Dentistry 20, no. 5 (2010): 366–373. 20545790 10.1111/j.1365-263X.2010.01054.x

[56] N. A. Aminabadi , A. Sohrabi , S. G. Oskouei , and B. A. Ajami , “Can Birth Order Affect Temperament, Anxiety and Behavior in 5 to 7‐year‐old Children in the Dental Setting?,” Journal of Contemporary Dental Practice 12, no. 4 (2011): 225–231. 22186855 10.5005/jp-journals-10024-1039

[57] F. Ghaderi , S. Fijan , and S. Hamedani , “How Do Children Behave Regarding Their Birth Order in Dental Setting?,” Journal of dentistry (Shiraz, Iran) 16, no. 4 (2015): 329–334. 26636121 PMC4664030

[58] T. M. Son , V. T. Nhu Ngoc , P. T. Tran , et al., “Prevalence of Dental Fear and Its Relationship With Primary Dental Caries in 7‐Year‐Old‐Children,” Pediatric Dental Journal 29, no. 2 (2019): 84–89.

[59] R. Sindhu , S. Rajaram , V. Bharathwaj , R. Mohan , S. Manipal , and D. Prabu , “Is Individual Deprivation Measures Associated With Dental Anxiety and Socioeconomic Status of Patients Visiting Dentists,” Indian Journal of Dental Research 31, no. 4 (2020): 515–519. 33107449 10.4103/ijdr.IJDR_802_18

[60] A. Krishnamurthy , S. Ranganath , M. Pramila , B. Chandrakala , and S. Sreekanteshwar , “Prevalence of Dental Fear in Children Belonging to Low Socio‐Economic Status‐A Cross Sectional Study in Bengaluru City,” Journal of Indian Association of Public Health Dentistry 9, no. 18 (2011): 177–181.

